# Integration of miRNA and Protein Profiling Reveals Coordinated Neuroadaptations in the Alcohol-Dependent Mouse Brain

**DOI:** 10.1371/journal.pone.0082565

**Published:** 2013-12-16

**Authors:** Giorgio Gorini, Yury O. Nunez, R. Dayne Mayfield

**Affiliations:** Waggoner Center for Alcohol and Addiction Research, The University of Texas at Austin, Austin, Texas, United States of America; CNRS UMR7275, France

## Abstract

The molecular mechanisms underlying alcohol dependence involve different neurochemical systems and are brain region-dependent. Chronic Intermittent Ethanol (CIE) procedure, combined with a Two-Bottle Choice voluntary drinking paradigm, represents one of the best available animal models for alcohol dependence and relapse drinking. MicroRNAs, master regulators of the cellular transcriptome and proteome, can regulate their targets in a cooperative, combinatorial fashion, ensuring fine tuning and control over a large number of cellular functions. We analyzed cortex and midbrain microRNA expression levels using an integrative approach to combine and relate data to previous protein profiling from the same CIE-subjected samples, and examined the significance of the data in terms of relative contribution to alcohol consumption and dependence. MicroRNA levels were significantly altered in CIE-exposed dependent mice compared with their non-dependent controls. More importantly, our integrative analysis identified modules of coexpressed microRNAs that were highly correlated with CIE effects and predicted target genes encoding differentially expressed proteins. Coexpressed CIE-relevant proteins, in turn, were often negatively correlated with specific microRNA modules. Our results provide evidence that microRNA-orchestrated translational imbalances are driving the behavioral transition from alcohol consumption to dependence. This study represents the first attempt to combine *ex vivo* microRNA and protein expression on a global scale from the same mammalian brain samples. The integrative systems approach used here will improve our understanding of brain adaptive changes in response to drug abuse and suggests the potential therapeutic use of microRNAs as tools to prevent or compensate multiple neuroadaptations underlying addictive behavior.

## Introduction

Cerebral cortex (CTX) and midbrain (MB) are two brain regions particularly susceptible to the effects of long-term alcohol abuse. CTX is prone to most severe brain damage in alcoholic human brain [1]–[3], has major connections with the mesolimbic reward pathway [4], [5], and is crucial for cognitive, executive and other important functions that are impaired in alcoholics [6], [7]. The development of alcoholism involves molecular alterations within the brain's reward neuronal circuits [8]–[10], and MB with its subdomains is well known to comprise addiction-related pathways which are crucial for drug responses.

Synergetic molecular interactions regulate neurobiological events associated with complex traits responsible for alcohol dependence. Although lists of candidate molecules have been reported based on their altered expression levels in alcohol-related studies [11], [12], their use is limited given the lack of contextual information and the documented low and inaccurate correspondence between transcript and protein levels [13]–[17]. Moreover, even small changes in the levels of certain microRNAs (miRNAs), which finely orchestrate gene and protein expression, could extensively impact brain function. For these reasons, alcohol researchers have recently started to focus on miRNAs in order to develop a more integrated profile of the effects of alcohol abuse [18].

miRNAs, short noncoding RNA molecules, have been effectively described as master regulators of the cellular transcriptome and proteome [19]. They can regulate their target genes in a cooperative, combinatorial fashion, where a single miRNA can target multiple mRNA transcripts and distinct miRNAs can target the same mRNA, ensuring fine tuning and control over a large number of cellular functions [20]. miRNAs are capable of inhibiting protein synthesis both by repressing translation and by facilitating deadenylation and subsequent degradation of mRNA targets [21]. In certain cases, miRNAs have even been reported to activate translation of targeted mRNAs [22]. miRNAs have been proposed as novel diagnostic biomarkers of human disease in circulating fluids such as plasma/serum [23], and there is recent evidence that they can act as signals for membrane receptor activation [24].

Previous microarray studies in human alcoholics and animal models have shown miRNA regulation. Differential miRNA expression has been reported following chronic intermittent ethanol exposure and withdrawal in primary cortical neuronal cultures [25], and persistent, coordinated changes in the expression of miRNAs and their target mRNAs have also been shown in rat medial prefrontal cortex following an alcohol dependence paradigm [26]. Furthermore, miRNA is up-regulated in the frontal cortex of human alcoholics who spent most of their adult lives consuming high quantities of ethanol without developing complicated alcohol-related disorders, indicating a robust homeostatic adaptation to the effects of alcohol [27]. Interestingly, this miRNA up-regulation might explain the down-regulation of certain genes in human alcoholic cases [28]–[32]. Indeed, recent evidences suggest that alcohol dependence results from changes in co-regulation that might not be detectable using single molecular-based analysis, since no one approach can fully account for the repercussion of individual changes on the complex interactions that regulate brain function. A number of studies have shown the interconnectedness among different stages of information processing at the molecular level. Quantitative proteomics studies have shown that miRNAs participate in fine-tuning the production of their targets, both at the mRNA and the protein level [33], [34]. Select clusters of gene expression profiles identified by array studies can be used to predict meaningful networks of interacting proteins [35]–[37]. Several components of protein complexes may be regulated simultaneously by a single miRNA or by several coexpressed miRNAs, and miRNAs that target the same protein complexes are frequently coexpressed [38]. Finally, several studies have demonstrated that the targets of single miRNAs are more connected in the protein-protein interaction (PPI) network than expected by chance [39]–[41].

Since miRNAs may regulate their targets at the translational level, without affecting mRNA abundance, the use of proteomic techniques is crucial to identify miRNA targets and to quantify the contribution of translational repression by miRNAs [42]–[45]. Our group has recently reported the differential regulation in cerebral cortex (CTX) and midbrain (MB) proteomes from C57BL/6J mice subjected to a chronic intermittent ethanol (CIE), two bottle choice (2BC) paradigm [46], which represents one of the best currently available animal models for alcohol dependence and relapse drinking. Here, we investigated the changes in global miRNA expression levels from the same brain samples and integrate the two datasets to investigate the molecular mechanisms of miRNA direct translational control during alcohol dependence. Novel systems-biology approaches have been utilized to comprehensively examine brain alterations in human alcoholics [47]. To improve our current molecular model of addiction, we used a systems approach to data analysis that combines miRNA and protein differential expression, miRNA and protein coexpression networks, miRNA target predictions, PPIs, and gene annotations.

## Materials and Methods

### Animals and Ethics Statement

Brain samples of male C57BL/6J mice subjected to Chronic Intermittent Ethanol paradigm combined with Two-Bottle Choice ethanol voluntary consumption were provided by Dr. Amanda J. Roberts (The Scripps Research Institute, La Jolla, CA), as previously described [46]. All procedures were conducted in accordance with the guidelines established by the National Institutes of Health in the Guide for the Care and Use of Laboratory Animals and were approved by The Scripps Research Institute's Animal Care and Use Committee (protocol: 11-0026). The paradigm used is summarized in Figure S1 and was based on earlier reports [48], [49]. Blood alcohol concentrations (BACs) were in the range shown to be critical for escalated ethanol drinking [50]. Brains were collected 72 hours after the last drinking session. CTX and MB were dissected from 7 CIE-2BC ethanol vapor-exposed (alcohol-dependent, high drinkers) mice, 7 Air-2BC air-exposed matched controls (which have also had access to alcohol), plus 7 ethanol-Naïve mice.

### miRNA expression analysis

Total RNA was isolated from the same 21 CTXs and 21 MBs analyzed in our previous study [46] using mirVana PARIS kit (Life Technologies, Carlsbad, CA), according to the manufacturer's instructions. Yield and quality of the RNA was determined using a 2100 Bioanalyzer (Agilent, Palo Alto, CA). Microarray hybridization was performed at the Moffitt Cancer Center microarray facility (Tampa, FL). Total RNA was hybridized with miRCURY 6th generation LNA™ microRNA arrays (Exiqon, Vedbaek, Denmark) to assess miRNA expression. The arrays included 1,223 human, 1,055 mouse, and 680 rat probes as referenced by miRBase v.16, in addition to many proprietary probes. Samples were labeled with Cy3, while the red channel was used for quality control and reference purposes. Images were analyzed using Imagene 8.0 (BioDiscovery, Hawthorne, CA). miRNA microarray data analysis was implemented in R environment using the Linear Models for Microarray Data (LIMMA) [51] and the Weighted Gene Correlation Network Analysis (WGCNA) [52], [53] packages from Bioconductor (http://www.bioconductor.org).

Data preprocessing included between-arrays quantile normalization [54] of single (green) channel, removal of flagged spots, and weighting. Background subtraction was not necessary. For each miRNA, a median was calculated from the intensity values of four replicates. Data were fitted into a linear model with an appropriate design matrix. Statistical differences between groups were calculated using an empirical Bayesian approach. False discovery rate (FDR) was assessed using the method of Benjamini and Hochberg [55]. Microarray data have been deposited online at NCBI Gene Expression Omnibus (GEO [56], accession number GSE48576).

### Protein expression analysis

Two-dimensional differential in-gel electrophoresis (2D-DIGE) was previously used to measure protein expression levels from the same 21 CTX and 21 MB samples. A comparative cross analysis of 28 gels (14 for CTX and 14 for MB samples) was performed as described [46], [57]. Briefly, 2,369 spots were detected and matched, and protein abundance values for individual samples as well as differential expression ratios between experimental groups were calculated. The standardized log abundances from 1,255 gel spots with at least 69/84 appearances were used for protein coexpression analysis. Based on significance, fold change, correlations, and appearances 93 spots were selected and identified by MALDI tandem mass spectrometry (MS) (Figure S2). Related MS data have been deposited to the ProteomeXchange Consortium via the PRIDE partner repository (dataset PXD000349, DOI 10.6019/PXD000349). The resulting protein coexpression dataset was integrated with miRNA coexpression data from the same samples in the present study.

### Weighted Gene Correlation Network Analysis (WGCNA)

*General information and purpose.* WGCNA is a bioinformatics tool which identifies significant over-represented patterns of directional changes in expression levels, consistently repeated across all the samples studied. We have previously used WGCNA to analyze protein coexpression [46]. In the present study, WGCNA was applied to miRNA expression data from the same samples. The correlation between miRNAs measures the degree of similarity between their expression patterns, and linkage hierarchical clustering can be used to detect modules, which are groups of interconnected miRNAs showing over-represented patterns of coexpression. We used WGCNA to examine modules of interconnected miRNAs, as well as proteins, showing over-represented patterns of coexpression. Module Eigengenes (MEs) summarize and represent each module in one synthetic expression profile. We used MEs to treat modules as single units and relate them to external information used as trait (CIE- and Air-2BC phenotypes) via simple measures (correlation).

miRNA microarray normalized intensity data were subjected to coexpression analysis implemented in R environment using “WGCNA” package from Bioconductor. Expression data for 1,023 miRNAs were used.

*Analytical procedure.* The general framework of WGCNA has been described in detail previously [47], [52]. We ran separate analyses for miRNAs in each region, as we have previously done with proteins [46]. First, a measure of similarity between the miRNA expression profiles was defined. This similarity measures the level of concordance between miRNA expression profiles across samples. Pearson's correlations were calculated for all pairs of miRNAs, and then a signed similarity (S_ij_) matrix was derived from S_ij_ = (1 + cor(x_i_,x_j_))/2, where expression profiles x_i_ and x_j_ consisted of the expression of miRNAs “i” and “j” across multiple microarray samples. In the signed networks, the similarity between miRNAs reflects the sign of the correlation of their expression profiles. Next, the similarity matrix was transformed into a weighted adjacency matrix of connection strengths by using a power adjacency function. The adjacency function (a_ij_) depends on certain parameters, which determine the sensitivity and specificity of the pairwise connection strengths. The signed similarity (S_ij_) was raised to power β (soft thresholding) to represent the connection strength (a_ij_): a_ij_ = S_ij_^β^. This step aims to emphasize strong correlations and reduce the emphasis of weak correlations on an exponential scale [53]. We chose the appropriate “Soft Power” (β  = 10) so that the resulting networks exhibited approximate scale-free topology (the lowest power for which the scale-free topology fit index Rˆ2>0.8), according to the criterion proposed by Zhang and Horvath [52]. Next, a topological overlap matrix (TOM) that measures the relative inter-connectedness of a pair of miRNAs in the network was built from the adjacency matrix, and the corresponding dissimilarity was obtained as d_ij_(a)≡1 − a_ij_. Average linkage hierarchical clustering of the topological overlap dissimilarity matrix was then used to identify clusters of co-expressed miRNAs (modules) [52]. miRNA modules correspond to branches of the hierarchical clustering tree (dendrogram). The resulting miRNA dendrogram was used for module detection with the “Dynamic Tree Cut” method: a “cutting height” of 0.995 with the preset “deep split” option ( = 2) were chosen to cut branches off the tree, and the resulting branches correspond to miRNA modules. The “minimum module size” parameter was set to limit the size of the smallest modules to at least 5 miRNAs, in order to avoid a large number of modules. The branches cut off of the miRNA tree corresponding to modules were labeled in unique colors. Unassigned miRNAs were labeled in gray.

*Relating modules to sample information.* In our analysis, we related modules to CIE paradigm. As a trait, the “Escalation of Consumption” (EoC) trait was intended as increased ethanol consumption, with “0” for the Naïve group, “1” for Air-2BC, and “2” for CIE-2BC. We also used actual average ethanol drinking amounts for the last 5-days 2BC session, plus miRNA expression and protein expression information from the same samples [46] as traits. miRNAs whose coexpression was highly and significantly correlated with the EoC trait were used as a trait for protein coexpression modules and *vice versa*.

### Real Time PCR analysis

Single-stranded cDNA was synthesized from total RNA using the TaqMan™ miRNA Reverse Transcription (Applied Biosystems, Foster City, CA). Following reverse transcription, quantitative RT-PCR (qRT-PCR) was performed in triplicate using TaqMan™ miRNA Assays (P/N: 4427975, Applied Biosystems) according to the manufacturer's instructions. All 7 samples for each experimental group were included in every reaction. The identification numbers for the single assays used are indicated in Table 1.

**Table 1:** Results of qRT-PCR analysis.

Comparison	microRNA	Exiqon probe ID	Array p-value	Array FC	TaqMan assay ID	RT-PCR p-value	RT-PCR FC	Ref. genes
CTX, CIE-2BC/Naïve	miR-488-3p	17316	6.47E-07	1.237	001659	3.36E-03	1.267	A, B, C
CTX, CIE-2BC/Naïve	miR-410-3p	11102	1.07E-04	1.178	001274	4.66E-03	1.241	A, B, C
CTX, CIE-2BC/Naïve	miR-3084-3p	148484	8.84E-07	1.284	461806_mat	7.19E-03	1.416	A, B, C
CTX, CIE-2BC/Naïve	let-7a-2-3p	42530	1.95E-04	0.772	463508_mat	9.84E-01	1.002	A, B, C
CTX, CIE-2BC/Naïve	miR-200a-3p	11000	1.27E-02	2.295	000502	1.40E-01	2.593	B, C
CTX, CIE-2BC/Naïve	miR-140-3p	42630	4.96E-02	0.927	002234	1.09E-02	0.572	B, C
CTX, CIE-2BC/Naïve	miR-141-3p	10946	8.56E-03	2.017	000463	2.85E-01	2.546	B, C
CTX, CIE-2BC/Naïve	miR-96-5p	13147	3.17E-03	1.849	000186	1.40E-01	2.622	B, C
CTX, CIE-2BC/Air-2BC	miR-3107-3p	42946	4.58E-02	2.352	462536_mat	3.96E-02	1.404	A, B
CTX, CIE-2BC/Air-2BC	miR-34b-5p	29153	8.87E-02	1.153	002617	1.66E-01	1.537	A, B
CTX, CIE-2BC/Air-2BC	miR-410-3p	11102	1.83E-02	1.098	001274	6.99E-01	1.11	A, B

Confirmation of differential expression for selected miRNA with real-time PCR. Total RNA from cortex samples was used, and all 7 samples for each experimental group were included (number of datapoints per subgroup, n = 7). Array p-values are based on a Bayesian two-tailed t-test, and TaqMan assays p-values are based on an unpaired t-test, corrected for multiple testing. Data were normalized to the average of the endogenous control genes indicated, based on qbasePLUS software's M scores. **A**, snoRNA142 (TaqMan assay ID: 001231); **B**, snoRNA234 (001234); **C**, U6 snRNA (001973). *ID*, Identification number; *FC*, fold change. *P-values in italics*, p<0.05.

qRT-PCR was carried out in a ViiA™ 7 Real-Time PCR System (Applied Biosystems), data collected using ViiA™ 7 Software v. 1.2.2 (Applied Biosystems), and qRT-PCR results imported into qbasePLUS software v. 2.4 (Biogazelle, Zwijnaarde, Belgium) [58]. Data were normalized to the average of the best endogenous control genes based on their M scores calculated by the software (Table 1). Unpaired t-test with correction for multiple testing was used to assess statistical significance. Target correlation was calculated using Pearson correlation.

### Functional annotations and bioinformatics tools

Functional annotations of differentially expressed miRNAs were obtained by using Ingenuity Pathway Analysis (IPA) (Ingenuity Systems, www.ingenuity.com). IPA Target Filter module was used to associate detected miRNAs with experimentally observed and predicted mRNA targets encoding for the differentially expressed or coexpressed proteins identified with 2D-DIGE and mass spectrometry from the same samples [46]. Target information data were filtered by considering the following: for differential expression data, miRNA p<0.06 (CTX) or p<0.05 (MB) and proteins p≤0.2 (CTX and MB); for coexpression data, miRNA and proteins p≤0.01 (CTX) or p<0.05 (MB) and correlation ≥0.5 (CTX and MB) (Table S1).

Integrative networks were built by combining our differential expression and coexpression data with miRNA target predictions obtained from IPA database and known/predicted PPIs from the Search Tool for the Retrieval of Interacting Genes/Proteins (String) database v.9.05 (confidence score: 0.15, http://string-db.org/). Collected information was loaded on Cytoscape v.2.8.3 (http://www.cytoscape.org/), and networks were generated and analyzed with several topology-based scoring methods [59]–[63].

## Results

### miRNA differential expression

**Figure 1:**
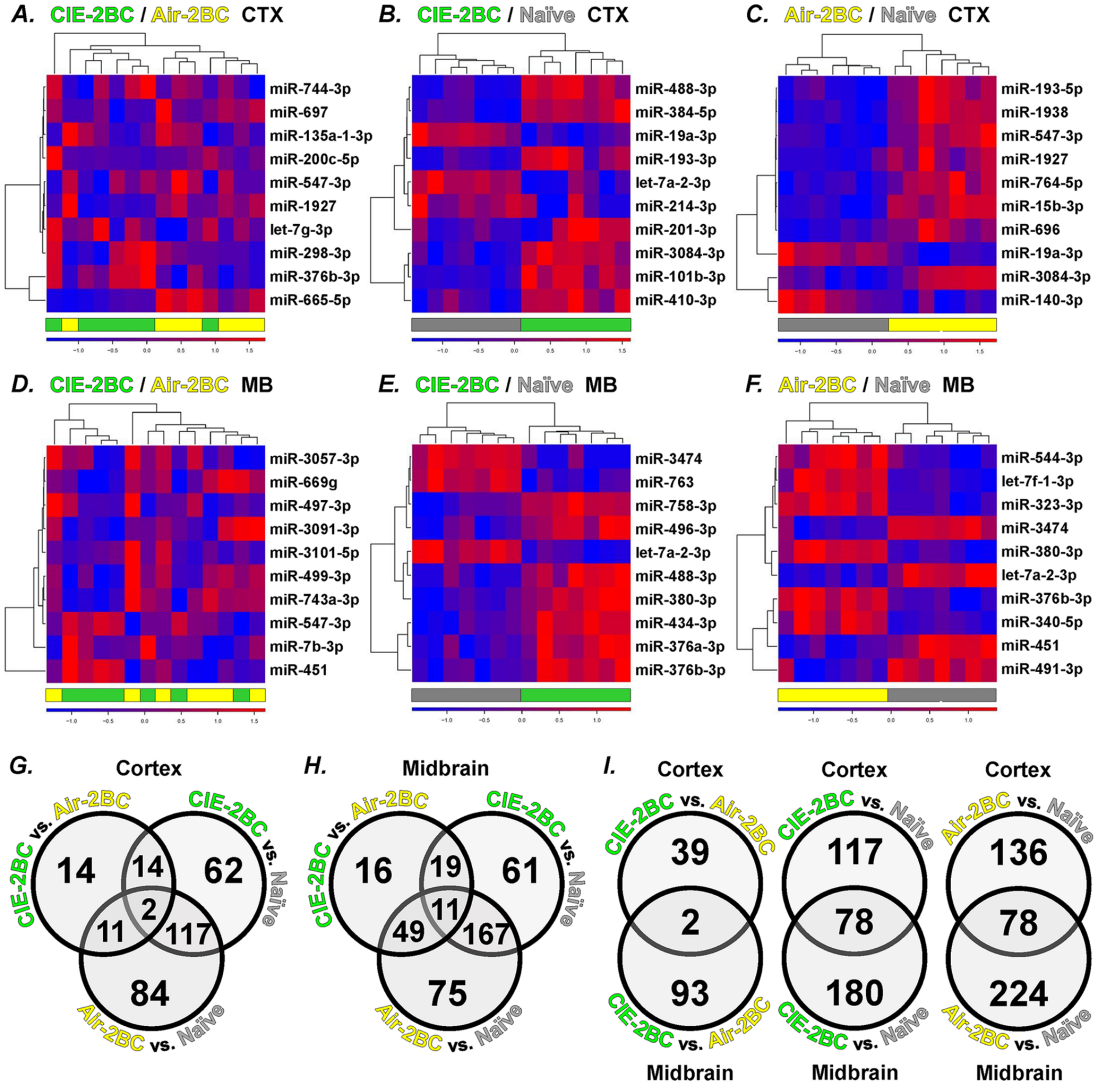
Hierarchical clustering of differentially expressed miRNAs from CIE-2BC (*green*), Air-2BC (*yellow*), and Naïve (*grey*) mice. Top 10 significant differentially expressed miRNAs for each comparison and brain region analyzed are shown. A-C, CTX; D-F, MB. A, D: CIE-2BC vs. Air-2BC; B, E: CIE-2BC vs. Naïve; C, F: Air-2BC vs. Naïve. *Rows*: individual miRNAs; *columns*: individual samples. Red within the heatmap represents miRNA up-regulation, and blue within the heatmap represents miRNA down-regulation. Each heat map shown contains 10 miRNAs with significant differential expression (p<5E-02, p<5E-04, p<5E-04, p<5E-03, p<5E-06, p<5E-07, respectively). Refer to Dataset S1 for p-values and fold change for individual miRNAs. The Venn diagrams indicate the number of shared and unique differentially expressed miRNAs among comparisons: between CIE-2BC versus Air-2BC, CIE-2BC versus Naïve, and Air-2BC versus Naïve groups in CTX (G), MB (H), and across the two brain regions (I). Only differences greater than 1.05 fold with p<0.05 (Bayesian two-tailed t-test) on mapped miRNAs eligible for IPA dataset filter are listed in Venn diagrams.

miRNA microarrays were used to measure expression profiles in the CTX and MB of mice subjected to CIE-2BC or Air-2BC and alcohol-naïve mice. The 2BC paradigm induced significant changes in miRNA levels with the most significant differences between the 2BC treated mice versus the Naïve group (Dataset S1). When comparing CIE-2BC to Naïve, approximately 200 miRNAs were differentially expressed in the CTX (p<0.05, fold change between 5 and 130%), and about 260 miRNAs were differentially expressed in MB (p<0.05, fold change between 5 and 120%). When comparing Air-2BC to Naïve, approximately 210 miRNAs were differentially expressed in the CTX (p<0.05, fold change between 5 and 118%), and 300 miRNAs were differentially expressed in the MB (p<0.05, fold change between 5 and 89%). The relative heatmaps for the top 10 differentially expressed miRNAs show complete group separation (Figure 1 B-C, E-F).

The differences were smaller and less significant when comparing CIE-2BC mice with their Air-2BC matched controls. Forty-one miRNAs were differentially expressed in the CTX (p<0.05) with a fold change between 5 and 135% compared to 95 miRNAs in the MB (p<0.05) with a fold change between 5 and 46% (Dataset S1). The relative heatmaps for the top 10 differentially expressed miRNAs show incomplete group separation (Figure 1 A, D).

### Over-represented functional categories

Top IPA functional categories for differentially expressed miRNAs between CIE-2BC and Air-2BC mice in CTX and MB include the following: cell cycle, endocrine system disorders, and inflammatory response (Table S2 A, D). Similar IPA analyses were carried out for other group comparisons in CTX and MB (Table S2 B-C, E-H). A comparison between regions shows greater adaptations in endocrine, hematological, and inflammatory disorders in the MB, with more differentially expressed miRNAs involved in related functional categories (Table S2 I). About ∼10% of differentially expressed and coexpressed miRNAs are predicted to target genes related to immune response, in both CTX and MB.

### miRNA-protein overall changes

The overall significant directional changes in miRNA and protein expression, for each comparison and brain region, are summarized in Table 2. When comparing CIE-2BC versus the Naïve group, a predominant miRNA up- and protein down-regulation was observed. On the contrary, when comparing CIE-2BC with their Air-2BC matched controls, an overall miRNA down- and protein up-regulation was prevalent.

**Table 2:** Global directional changes in miRNA and protein expression induced by CIE paradigm.

Brain Region	Comparison	Molecule type	N. DE	% DE	% ↑	% ↓	Prevalent Direction	Paired Exp.
CTX	A. CIE-2BC vs. Air-2BC	miRNAs	45	4%	44%	47%	↓	↓↑
		proteins	54	2%	52%	43%	↑	
CTX	B. CIE-2BC vs. Naïve	miRNAs	219	21%	59%	36%	↑	↑↔
		proteins	232	10%	45%	46%	↔	
CTX	C. Air-2BC vs. Naïve	miRNAs	237	23%	55%	38%	↑	↑↑
		proteins	344	15%	49%	44%	↑	
MB	D. CIE-2BC vs. Air-2BC	miRNAs	108	11%	30%	58%	↓	↓↑
		proteins	57	2%	61%	28%	↑	
MB	E. CIE-2BC vs. Naïve	miRNAs	321	31%	62%	31%	↑	↑↓
		proteins	175	7%	43%	48%	↓	
MB	F. Air-2BC vs. Naïve	miRNAs	376	37%	65%	24%	↑	↑↓
		proteins	226	10%	37%	53%	↓	

Overall directional changes in miRNA (microarrays) and protein spot (2D-DIGE) expression for each comparison and brain region analyzed. Comparisons **A-F** are named as in Figure 1. *N. DE*, number of differentially expressed molecules with fold change >5% or <-5% and p<0.05 (gel appearances were not considered in this case); *% DE*, percentage of differentially expressed molecules based on their total number (1,023 miRNAs and 2,369 protein spots detected); % *↑* and % *↓*, percentages of up- and down-regulated molecules; *Paired Exp.*, paired expression between miRNAs and protein spots in the same brain region and comparison.

### WGCNA analysis

**Figure 2:**
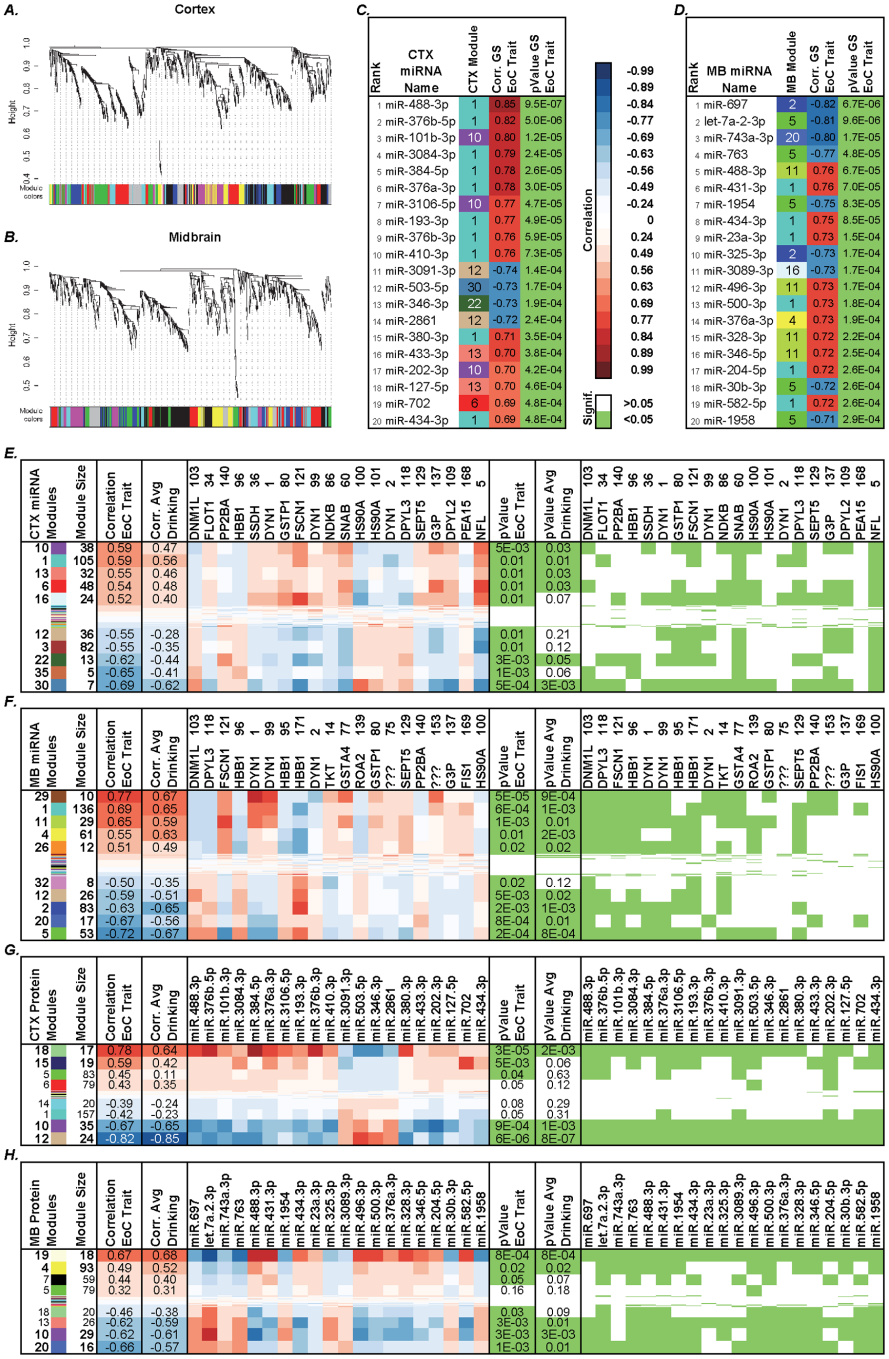
WGCNA analysis of miRNA expression in CTX and MB of mice subjected to CIE paradigm identified distinct modules of coexpressed miRNAs. A and B show dendrograms produced by average linkage hierarchical clustering. Horizontal color bars represent different coexpression modules. Bar sizes correspond to the number of miRNAs in each module. SoftPower β  = 10 (A), 9 (B), minModuleSize = 5, cutHeight = 0.995, deepSplit = 2 (see *Methods*). Tables C, D show the relative contribution of miRNAs to CIE paradigm, in terms of correlation (*Corr.*) between the individual top 20 coexpressed miRNAs sorted by their gene significance (*GS*) for the EoC trait, with relative p-values and rank. Module number and color information are also included. Full lists are reported in Dataset S1. Tables E-H show correlation between modules of coexpressed miRNAs (E, F) or proteins (G, H) and the EoC trait, or the average 2BC ethanol consumption, as well as individual proteins or miRNAs as traits. Modules are named by a number and a color. Protein names are followed by corresponding gel spot number. In correlation columns, blue represents negative and red represents positive correlations, as reported on the legend. Green p-values are <0.05. Part of the data shown in G and H is taken from our previous study [46].

WGCNA analysis was performed on normalized expression data from 1,023 miRNAs. Average linkage hierarchical clustering identified 39 distinct modules of coexpressed miRNAs in the CTX and 39 in the MB (Figure 2A, B). We related MEs (see *Methods*) to CIE paradigm phenotypic data (“EoC” and drinking) used as trait through correlation analysis. In both regions, some modules are highly correlated with the 2BC EoC trait and the average drinking (for the last 5-days 2BC session). miRNA modules CTX10, CTX1, MB29, MB1, and MB11 were highly positively correlated (corr. = 0.58-0.69), while modules CTX30, CTX35, CTX22, MB5, MB20, and MB2 were highly negatively correlated (corr.<-0.6) to the EoC trait (Figure 2E, F). A list of the top 20 significantly coexpressed miRNAs is shown in Figure 2C, D.

Our previous protein WGCNA analysis was integrated with the present miRNA coexpression analysis. Data from individual CIE-related proteins, as well as information from 19 protein coexpression modules in the CTX and 23 in the MB (Figure 2G, H) were used.

Individual proteins whose coexpression was highly and significantly correlated with the EoC were used as a trait for miRNA coexpression modules, and top correlated individual miRNAs were used as a trait for protein modules. Several proteins were highly negatively correlated with miRNA modules important (positively correlated) for the EoC trait (i.e., distinct isoforms of DNM1L, HBB1, HS90A, DYN1, Figure 2E, F). Similarly, several miRNAs were highly negatively correlated with protein modules that in turn are positively correlated to the EoC trait, and they often belonged to the same miRNA module (i.e., miR-3091-3p and miR-2861 in CTX and let-7a-2-3p and miR-763 in MB, Figure 2G, H).

### Validation by Real Time PCR analysis

To validate results from miRNA microarray analysis, we performed qRT-PCR. A representative subset of 10 significantly differentially regulated miRNAs was tested. One non-significantly regulated miRNA was also tested as control. Following qRT-PCR, five miRNAs were significantly regulated in the same direction as shown by the microarray analysis (Table 1). Five other miRNAs did not achieve statistically significant differences, but in 4 cases we were still able to detect the expected change in expression levels. The non-significantly regulated miRNA did also not achieve statistical significance when tested by qRT-PCR.

Furthermore, when comparing the qPCR expression patterns of the different miRNAs tested across the 14 samples used, miR-200a-3p showed a 0.98 Pearson correlation (p = 3.6E-8) with miR-96-5p and 0.97 correlation (p = 1E-7) with miR-141-3p. Indeed, these three miRNAs were identified as coexpressed by WGCNA analysis, belonging to the same miRNA module (CTX23).

### Integrative networks

**Figure 3:**
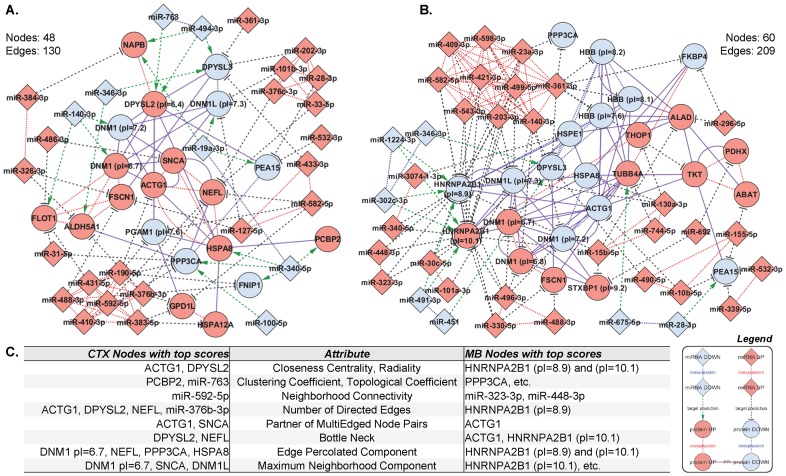
Integrative networks for CTX and MB. To highlight molecular mechanisms underlying the transition from alcohol consumption to dependence in the CIE paradigm, we integrated information from miRNA and protein differential expression and coexpression analyses with currently available miRNA target predictions (IPA database), as well as known and predicted PPIs (String database). The resulting networks for CTX (**A**) and MB (**B**) were analyzed with several topology-based scoring methods (**C**). Nodes with highest score for the corresponding network attribute are listed. *Blue*, down-regulation; *red*, up-regulation; *diamonds*, miRNAs; *circles*, mRNAs (genes encoding for the identified proteins); *dashed lines*, target predictions; *continuous lines*, PPIs; *dotted lines*, coexpression. Refer to *Methods* for further details.

To distinguish key molecules involved in the escalation of ethanol consumption to dependence in the CIE paradigm, we integrated information from miRNA and protein differential expression and coexpression analyses with currently available miRNA target predictions (IPA database, Table S1), as well as known and predicted PPIs (String database). The resulting networks involve 48 (CTX) and 60 (MB) molecular nodes, featuring several diverse regulatory mechanisms: coexpressed miRNAs targeting the same regulated gene (e.g., miR-532-3p and miR-339-5p on *Pea15* in the MB), genes encoding physically interacting or coexpressed proteins targeted by the same miRNA (e.g., miR-494-3p on both *Dpysl2 and Dpysl3*, and miR-140-3p on coexpressed *Flot1* and *Dnm1* in the CTX), and isoform-specific translational repression (e.g., *Hnrnpa2b1* in the MB). Furthermore, miRNA coexpression modules CTX1, CTX10, CTX12, MB1, and MB26 regulate proteins in modules CTX3, CTX18, MB11. The resulting networks for CTX and MB were then analyzed with several topology-based scoring methods (Figure 3). Top-scoring molecular nodes include proteins encoded by genes *Actg1*, *Dnm1*, *Dnm1l*, *Dpysl2*, *Nefl*, *Ppp3ca*, and *Snca* in the CTX, and *Actg1*, *Hnrnpa2b1*, and *Ppp3ca* in the MB.

## Discussion

The global impact of miRNAs on protein output has been reported *in vitro* in cell cultures [33], [34], [43], [64], and *ex vivo* from human cartilage [65] and mouse cochlea and vestibule [66], but ours is the first study to report opposing miRNA/protein expression from the same brain samples of alcohol dependent mice. Our integrated approach surpasses the gene- and protein-centric analyses that have become popular in alcohol research. This is an important distinction, since mRNA expression does not always reflect the actual protein levels [13]. By combining two high-throughput techniques, we obtained a comprehensive identification and characterization of molecular events associated with alcohol consumption and dependence. While our arrays contain the most up to date and complete mouse miRNA probe set, limitations in 2D-DIGE technology include reproducibility and relatively small dynamic range.

As previously reported for proteins, we observed smaller net effects, as expected, when comparing differentially expressed miRNAs between CIE-2BC with air-matched controls since both groups had access to alcohol in the 2BC model. We identified diverse sets of deregulated miRNAs in CTX and MB and showed that distinct sets of miRNAs were associated with alcohol consumption versus dependence (Figure 1), suggesting critical neurobiological adaptive changes during the transition from alcohol consumption to dependence. This could be explained by the evidence that although both 2BC-exposed groups consumed increasing levels of ethanol, CIE-2BC were subjected to higher brain alcohol concentrations compared with Air-2BC mice and experienced signs of alcohol dependence. Notably, miRNA profiles from CIE-2BC versus Naïve animals segregated into clearly separable clusters (Figure 1). When comparing CIE-2BC versus Naïve mice, we found miRNA to be predominantly up-regulated while protein was predominantly down-regulated (Table 2). On the contrary, we found a prevalence of miRNA down-regulation and protein up-regulation when we compared CIE-2BC mice with their air matched controls in both CTX and MB.

MiRNA up-regulation has previously been shown in prefrontal cortex of human alcoholics who drank high alcohol doses for many years [27]. Oxidative stress and morphine treatment were also associated with increased miRNA expression *in vitro* and *ex vivo* [67], [68]. In addition, miRNA up-regulation has been reported in rat striatum following extended cocaine self-administration [69] and in mouse dopamine 2 receptor-expressing neurons after acute cocaine injections [70]. Conversely, miRNA down-regulation was prevalent in the medial prefrontal cortex of alcohol post-dependent rats [26]. In our current study, we found prevalent miRNA up-regulation in the CTX and MB when we compared CIE-2BC mice with alcohol-naïve animals. The resulting net down-regulation that we observed between CIE-2BC and Air-2BC mice (Table 2) may be due to expression levels of some miRNAs rising as an initial response to alcohol consumption (higher in Air-2BC group), but then slightly going back with the establishment of dependence in CIE-2BC (e.g., miR-497-3p in MB). However, other miRNA levels are increased or decreased in proportion to the amounts of alcohol consumed (as shown by WGCNA) or augmented in dependent mice only (e.g., miR-1955-5p and miR-486-3p in CTX). Such dynamicity in brain miRNA-mediated adaptations to the effect of alcohol has not been previously described on a global scale and provides intriguing evidence for the intricate role of miRNAs in the development of alcohol dependence. The dynamics of miRNA expression are thought to parallel the widespread changes in gene expression following excessive alcohol consumption, in agreement with the functional fine-tuning of miRNAs on the transcriptional regulatory network [33], [34]. Hence, the observed differences in miRNA levels of CIE-2BC dependent mice may compensate for, or reprogram, transcriptional regulatory processes. Additional differences between alcohol dependent (CIE-2BC) and non-dependent (Air-2BC) mice were found at the protein level due at least in part to translational inhibition by miRNAs, and this differential regulation might delineate the molecular signature of alcohol dependence. It is possible that even small individual changes in the identified miRNAs could exert cumulative effects by ultimately affecting proteins; a change in protein expression level following chronic ethanol could then alter the availability of proteins for important PPI networking and the composition of respective protein complexes, highlighting the overall impact of individual, small fold-changes among miRNAs and their relation to a molecular model of alcohol dependence. Our integrative systems approach offers a novel perspective on these complex remodeling mechanisms in response to alcohol consumption and dependence.

In our previous proteomic analysis from the same samples we reported isoforms of dynamin-1 as distinctively regulated in CTX and MB [46]. These isoforms showed an escalating up-regulation or a gradual down-regulation during the transition from alcohol consumption to dependence. We also showed evidence of significant up-regulation of *Dnm1* gene expression in the CTX by RT-PCR. Accordingly, we here report miR-140, which targets dynamin [71], as down-regulated in the CTX of both 2BC groups but conversely up-regulated in the MB. This could be explained by the onset of midbrain-specific adaptations related to reward neurocircuits; another possibility is that miR-140 targets different isoforms of dynamin in different brain regions.

**Figure 4:**
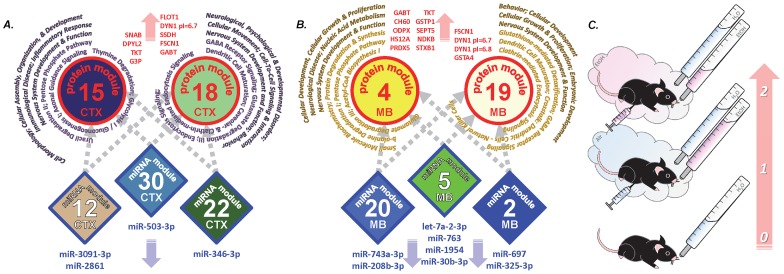
Provisional representation of miRNA-mRNA modular network derived from miRNA and protein WGCNA and correlation analysis in mouse CTX (A) and MB (B). Module colors reflect those from WGCNA analysis. Increasing ethanol consumption levels can be described as “0” for the Naïve group, “1” for Air-2BC, and “2” for CIE-2BC (EoC trait, sketched in C). Protein modules (*circles*) that are more relevant to alcohol actions are shown based on their correlation to the EoC trait. Proteins in these modules showed an increasing up-regulation across experimental groups, parallel to the EoC trait. miRNA modules (*diamonds*) negatively correlated to the EoC trait are also shown; the miRNAs in these modules showed an increasing down-regulation across experimental groups, inversely proportional to the EoC trait. These miRNAs are also oppositely correlated to the protein modules which are in turn important for the EoC trait. Therefore, miRNAs and proteins listed respond to 2BC drinking and/or CIE with opposite directional changes in their expression levels, and might thus play a crucial role in excessive ethanol drinking associated with dependence. Protein modules related to endocytic pathways and energy metabolism appear to be important for the effects caused by CIE paradigm. *Blue and red outline*, negative and positive correlation with the EoC trait.

We used a sophisticated systems approach to data analysis and applied WGCNA [53] to identify groups of interconnected miRNAs showing over-represented patterns of coexpression. Some of these distinct modules of coexpressed miRNAs (Figure 2E, F) showed a high correlation with the EoC trait and alcohol drinking in CTX and MB. To further investigate putative miRNA translational control mechanisms, we then linked proteins to the contextual miRNA information by using protein expression data from the same samples as a trait for the miRNA coexpression modules. We found that the miRNA modules which were best correlated with the EoC trait were also negatively correlated with some coexpressed and differentially expressed (down-regulated) proteins (e.g., DNM1L and HS90A in CTX, DNM1L, DPYL3, and DYN1 pI = 7.2 in MB) and positively correlated with other up-regulated ones (e.g., FSCN1, DYN1 isoforms in MB, etc.). In turn, we used miRNA coexpression data as a trait for the coexpressed protein modules and found that the protein modules that best correlated to the EoC trait were negatively correlated with down-regulated miR-3091-3p, miR-503-5p, 346-3p, miR-2861 in CTX (Figure 2G) and also negatively correlated with down-regulated let-7a-2-3p, miR-763, miR-1958, miR-697 (among others) in MB (Figure 2H). On the other hand, up-regulated miR-384-5p in CTX and miR-488-3p in MB were among the miRNAs with the highest positive correlations with protein modules associated with the EoC trait. Further inspection revealed that the individual miRNAs, oppositely correlated with the top protein modules, also belonged to miRNA coexpression modules which were highly, inversely correlated to the EoC trait (CTX12, CTX22, CTX30, MB2, MB5, and MB20) (Figure 2C, D and Dataset S1). Figure 4 depicts a miRNA-protein modular scenario representing a unique integrated model of alcohol drinking and dependence. Increasing levels of alcohol consumption leading to physical dependence (EoC trait) may involve a gradual synergistic down-regulation of the negatively highly correlated miRNAs mentioned above; the corresponding gradual looseness in their translational repression was reflected in an escalating two-step up-regulation of proteins important for energy metabolism and endocytic pathways in Air-2BC and CIE-2BC mice compared to Naïve. Indeed, protein modules CTX15, CTX18, MB4, and MB19 were highly, positively correlated to the EoC trait. At the same time, these protein modules were also positively correlated with certain coexpressed miRNAs (e.g., miRNA modules CTX1, CTX10, MB11, and MB1, see Figure 2G-H, C-D): since in this case miRNAs and proteins are regulated in the same direction, this may either involve an indirect translational control step via transcription factors or reflect a time lapse in the temporal evolution of gene network adaptive responses during disease progression that we could not capture with our experimental design.

The significance of our WGCNA analysis is verified by the RT-PCR experiments, where we tested the expression levels of three miRNAs from the same module and obtained high Pearson correlations among their expression patterns across the samples (Table 1). This highlights the complementarity of differential expression and coexpression for detecting coordinated molecular changes.

We generated a sophisticated integrative network to examine finely orchestrated molecular changes important for the transition from alcohol consumption to dependence, using currently available miRNA target predictions from the IPA database (Table S1) and PPIs from the String database along with information from differential expression and coexpression of miRNAs and proteins (Figure 3). In miRNA regulation there is often a connection between co-regulated entities; protein connectivity and miRNA regulation complexity are positively correlated [40], and a single miRNA or coexpressed miRNAs are likely to have cumulative effects by simultaneously targeting several components of protein complexes [38], [39], [41]. Remarkably, in our integrative network several target genes of differentially expressed miRNAs encode regulated components of interacting proteins complexes or coexpressed proteins.

**Figure 5:**
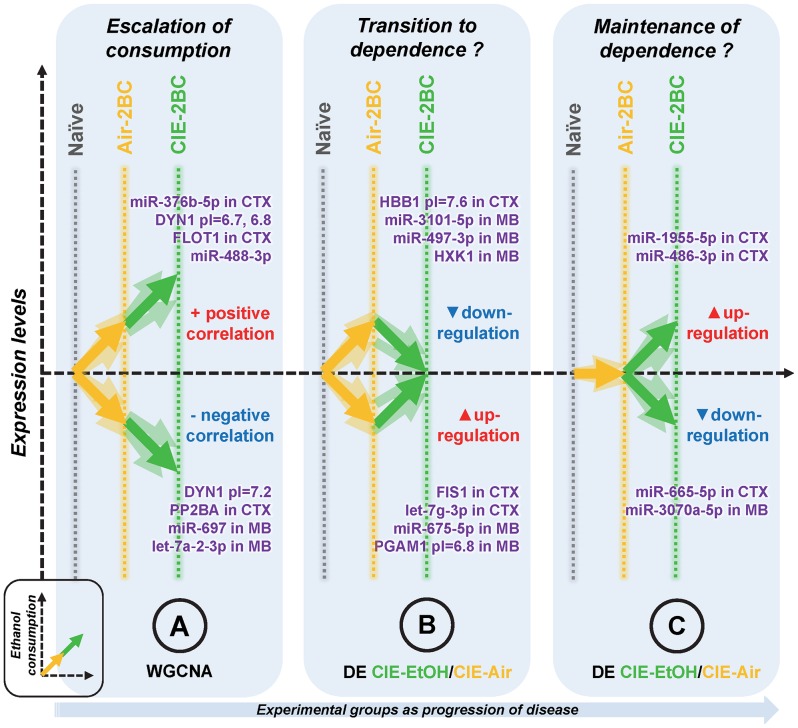
General classification of brain molecular changes underlying the escalation of ethanol consumption associated with dependence. We propose three overall patterns of change and list examples of molecules specific to each category (*in purple*). Y-axis represents the level of expression, and x-axis shows the progression of the disease, in terms of patterns of changes and experimental groups (*grey*, Naïve; *yellow*, Air-2BC; *green*, CIE-2BC). Changes associated with escalation of consumption (A) were identified by WGCNA analysis, and are positively (*red*) or negatively (*blue*) correlated with the amounts of alcohol consumed (*inset*); therefore, these modifications are more pronounced in the CIE-2BC than the Air-2BC group. Changes important for the transition to dependence (B) are almost exclusively present in Air-2BC but not CIE-2BC mice, compared to Naïve; The resulting CIE-/Air-2BC ratio from the differential expression analysis (*DE*) shows down- (*blue*) or up- (*red*) regulation, although the changes in Air-2BC follow the opposite direction. Finally, changes associated with the maintenance of alcohol dependence (C) include those molecules regulated in CIE-2BC but not in Air-2BC mice; their expression levels remain unmodified in non-dependent animals, and thus the differential expression ratio follows the same direction of the respective expression levels.

The molecular remodeling suggested in our study is summarized in Figure 5 and depicts how the brain can gradually regain stability through widespread neuroadaptations following alcohol exposure. miRNAs or proteins associated with increasing levels of alcohol consumption are regulated in the same direction in 2BC groups compared to Naïve (Figure 5A); however, these modifications are always more pronounced in the CIE-2BC than the Air-2BC group, since their expression levels are proportional (or inversely proportional) to the amounts of alcohol consumed, as shown by WGCNA analysis (i.e., modules highly positively/negatively correlated to the EoC trait). Indeed, such dysregulations become more severe with repeated alcohol consumption, and this in turn causes further alcohol intake. Another distinct set of molecular changes is represented by the miRNAs and proteins which are exclusively regulated in Air-2BC but not in CIE-2BC mice (Figure 5B), indicating their specific role during the transition to dependence. The directional changes in Air-2BC group are in this case opposite to what is the resulting CIE-2BC/Air-2BC differential expression. Furthermore, proteins and miRNAs regulated in CIE-2BC but not Air-2BC mice (Figure 5C) could be crucial for the maintenance of alcohol dependence, since their expression levels are unmodified in non-dependent animals. This conceptual perspective of our research is consistent with the allostatic model of drug addiction [72].

In summary, our integrative analysis of global miRNA and protein expression levels from different brain regions of mice subjected to a CIE paradigm uncovered coordinated, synergistic regulation of miRNAs and proteins with direct evidence of altered brain translational control driving the behavioral transition from alcohol consumption to dependence. The concomitant, opposite gradual molecular changes related to behavioral patterns provide a more comprehensive and integrated picture of the molecular basis of addiction, and suggests the potential therapeutic use of miRNAs as tools to prevent or compensate multiple neuroadaptations underlying addictive behavior.

## Supporting Information

Dataset S1 Changes in miRNA expression induced by CIE paradigm. All the detected miRNAs sorted by their p-value for differential expression in the comparison CIE-2BC vs. Air-2BC. Tables include differential expression (DE) for all the comparisons and brain regions analyzed. Red represents miRNA up-regulation and blue represents miRNA down-regulation. Data from WGCNA analysis are also included, providing the relative contribution of each miRNA to the effects of the EoC trait. WGCNA-related columns show correlation between individual miRNAs and the EoC trait, with relative p-values and rank. Module information is also included: number, color, and frequency (size). In correlation columns, blue represents negative and red represents positive correlations. Green p-values are <0.05.
(XLS)

Figure S1 General CIE protocol for 2BC drinking. Mice were made physically dependent on alcohol by intermittent EtOH vapor exposure (3X16h EtOH + 8h Air). The EtOH consumption was measured during the 2h limited access, 2BC procedure. The injections consisted of 68.1 g/kg pyrazole + saline or pyrazole + 1.5 g/kg 20% EtOH. Following the vapor/control chamber exposures (2BC#1 and 2BC#2) there were significant increases in EtOH consumption in CIE-2BC vapor-exposed mice relative to Air-2BC control mice. *p<0.05 post-hoc analysis. Group average levels of ethanol consumed are expressed in g/Kg. The figure is adapted from [46].
(TIF)

Figure S2 Representative 2D-DIGE gel used for proteomic experiments. Gel images were scanned immediately following the SDS-PAGE. Each scan revealed one of the CyDye signals (Cy2, Cy3 and Cy5). Cy2 was used to normalize the signals from Cy3 and Cy5 channels. Single and overlay images were generated to compare different samples, and a comparative analysis of all spots was performed using DeCyder “in-gel” or “cross-gel” analysis software. The overlay image shown was obtained from gel#14, CIE-2BC sample CTX#20 compared to the Air-2BC sample CTX#15. Spots of interest were selected based on 1.15-fold, allowing for the appearance of the spots in 23 out of 28 gels (69 out of 84 total images). The 93 spots shown were picked, trypsin digested, and subjected to MALDI-TOF MS and TOF/TOF tandem MS/MS; resulting peptide mass and the associated fragmentation spectra were submitted to MASCOT search engine. Candidates with either protein score C.I. % or Ion C.I. % greater than 95 were considered significant. The best matches were selected based on C.I.% and pI/MW location of the spot in the gel. Protein accession name is indicated for each spot. The figure is adapted from [46].
(TIF)

Table S1 miRNA target prediction analysis. IPA Target Filter module was used to associate detected miRNAs with experimentally observed and predicted mRNA targets encoding for the differentially expressed or coexpressed proteins identified with 2D-DIGE and mass spectrometry. Target information data were filtered by considering the following: for differential expression data, miRNA p<0.06 (CTX) or p<0.05 (MB) and proteins p≤0.2 (CTX and MB); for coexpression data, miRNA and proteins p≤0.01 (CTX) or p<0.05 (MB) and corr. ≥0.5 (CTX and MB).
(XLSX)

Table S2 Ingenuity Pathway “Core” analysis of differentially expressed miRNAs. IPA summary for differentially expressed miRNAs across multiple comparisons. **A-C**, **G**, CTX; **D-F**, **H**, MB. **A**, **D**: CIE-2BC vs. Air-2BC; **B**, **E**: CIE-2BC vs. Naïve; **C**, **F**: Air-2BC vs. Naïve. **G**, **H**: CIE-2BC/Air-2BC minus CIE-2BC/Naïve and Air-2BC/Naïve. miRNAs used for the analysis were selected from CTX and MB based on fold change ≥5% or ≤-5% and p<0.05. The following data are reported: top networks, top biological functions, listed with their respective scores, p-values, and number of molecules involved. The tab **I** shows a comparison between IPA analyses for CIE-2BC vs. Air-2BC in CTX and MB.
(XLSX)
