# Correction: Integration of miRNA and Protein Profiling Reveals Coordinated Neuroadaptations in the Alcohol-Dependent Mouse Brain

**DOI:** 10.1371/annotation/6286be0f-d729-495a-8a72-78995e9ceda7

**Published:** 2013-12-30

**Authors:** Giorgio Gorini, Yury O. Nunez, R. Dayne Mayfield

This Article was republished on December 23rd 2013 due to missing figures in the PDF. Please download the PDF again to view the correct figures. 

