# Supplementary figures and images for: Integration of miRNA and Protein Profiling Reveals Coordinated Neuroadaptations in the Alcohol-Dependent Mouse Brain
Source: PLoS One. 2013 Dec 16;8(12):e82565. doi: 10.1371/journal.pone.0082565 (PMC3865091; doi:10.1371/journal.pone.0082565)

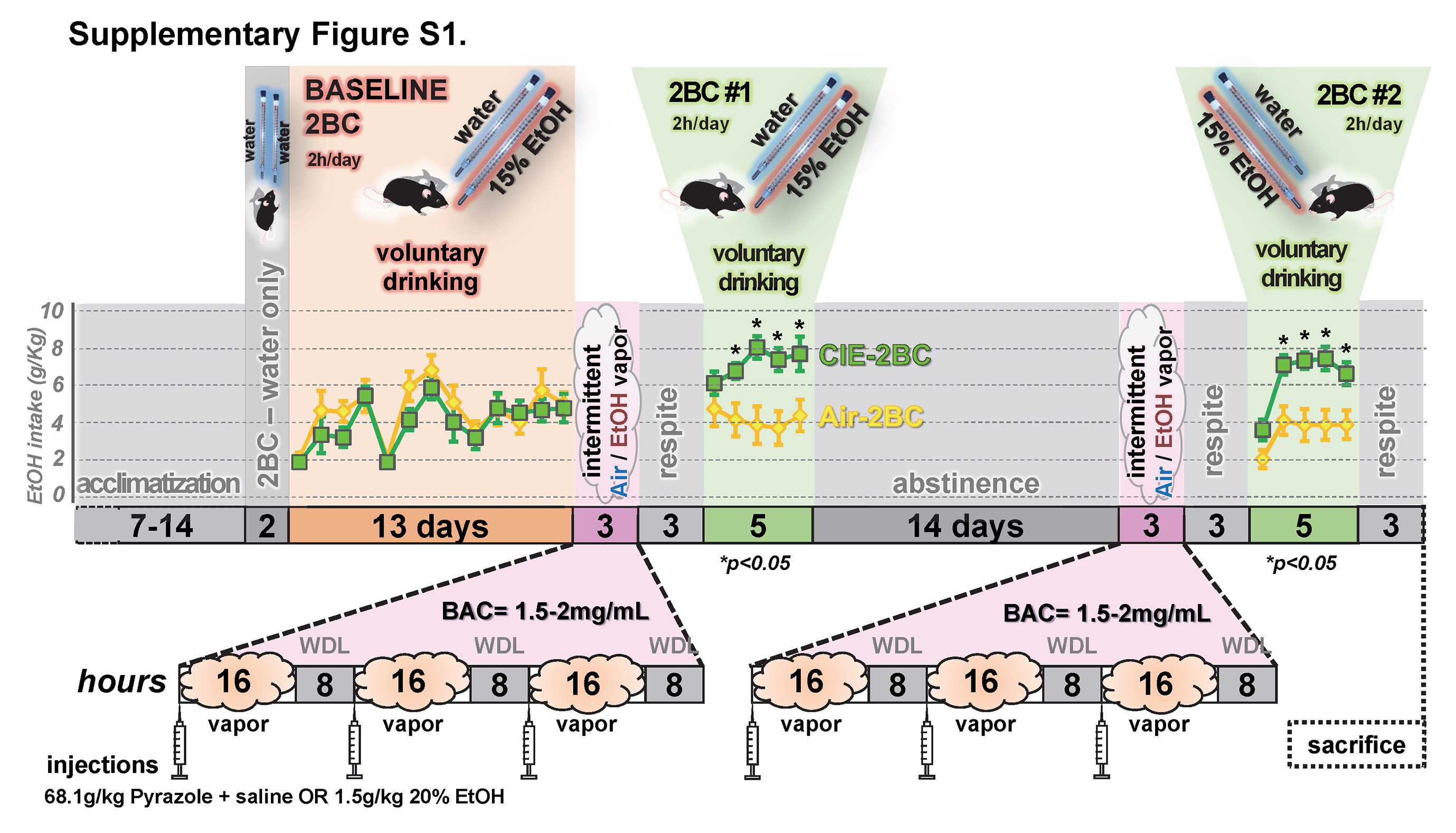

Supplement: Figure S1 — General CIE protocol for 2BC drinking. Mice were made physically dependent on alcohol by intermittent EtOH vapor exposure (3X16h EtOH + 8h Air). The EtOH consumption was measured during the 2h limited access, 2BC procedure. The injections consisted of 68.1 g/kg pyrazole + saline or pyrazole + 1.5 g/kg 20% EtOH. Following the vapor/control chamber exposures (2BC#1 and 2BC#2) there were significant increases in EtOH consumption in CIE-2BC vapor-exposed mice relative to Air-2BC control mice. *p<0.05 post-hoc analysis. Group average levels of ethanol consumed are expressed in g/Kg. The figure is adapted from [46]. (TIF) [file pone.0082565.s002.tif]

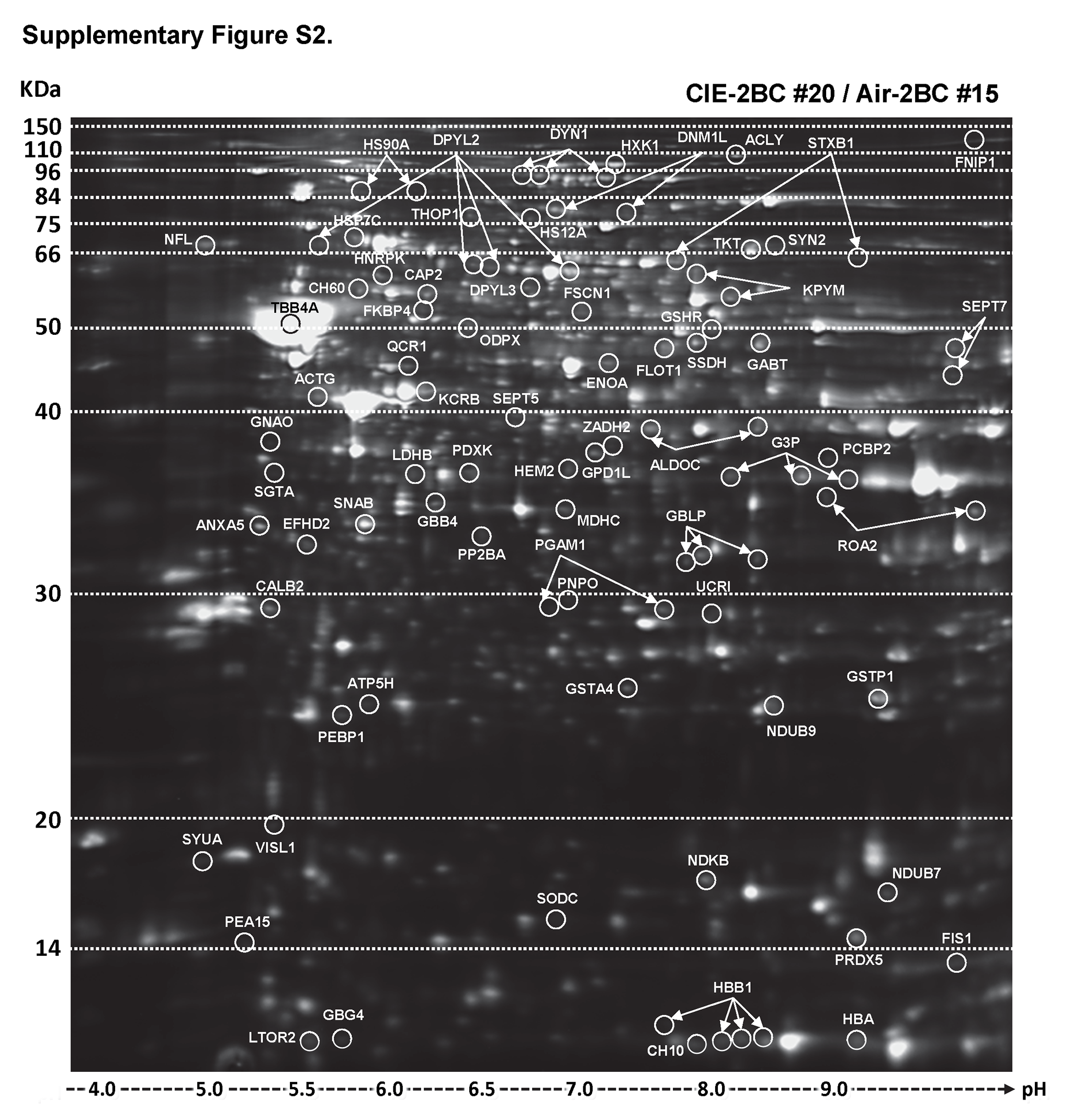

Supplement: Figure S2 — Representative 2D-DIGE gel used for proteomic experiments. Gel images were scanned immediately following the SDS-PAGE. Each scan revealed one of the CyDye signals (Cy2, Cy3 and Cy5). Cy2 was used to normalize the signals from Cy3 and Cy5 channels. Single and overlay images were generated to compare different samples, and a comparative analysis of all spots was performed using DeCyder “in-gel” or “cross-gel” analysis software. The overlay image shown was obtained from gel#14, CIE-2BC sample CTX#20 compared to the Air-2BC sample CTX#15. Spots of interest were selected based on 1.15-fold, allowing for the appearance of the spots in 23 out of 28 gels (69 out of 84 total images). The 93 spots shown were picked, trypsin digested, and subjected to MALDI-TOF MS and TOF/TOF tandem MS/MS; resulting peptide mass and the associated fragmentation spectra were submitted to MASCOT search engine. Candidates with either protein score C.I. % or Ion C.I. % greater than 95 were considered significant. The best matches were selected based on C.I.% and pI/MW location of the spot in the gel. Protein accession name is indicated for each spot. The figure is adapted from [46]. (TIF) [file pone.0082565.s003.tif]
